# An In-Vitro Evaluation of Microleakage in Resin-Based Restorative Materials at Different Time Intervals

**DOI:** 10.3390/polym14030466

**Published:** 2022-01-24

**Authors:** Afreen Bilgrami, Mohammad Khursheed Alam, Fazal ur Rehman Qazi, Afsheen Maqsood, Sakeenabi Basha, Naseer Ahmed, Kausar Ali Syed, Mohammed Mustafa, Deepti Shrivastava, Anil Kumar Nagarajappa, Kumar Chandan Srivastava

**Affiliations:** 1 Department of Dental Materials, Fatima Jinnah Dental College, Karachi 74900, Pakistan; afreenagha@hotmail.com; 2 Orthodontics, Department of Preventive Dentistry, College of Dentistry, Jouf University, Sakaka 72345, Saudi Arabia; 3 Department of Dental Research Cell, Saveetha Dental College and Hospitals, Saveetha Institute of Medical and Technical Sciences, Chennai 600077, India; 4 Department of Public Health, Faculty of Allied Health Sciences, Daffodil International University, Dhaka 1230, Bangladesh; 5 Department of Operative Dentistry, Dr. Ishrat-Ul-Ebad Khan Institute of Oral Health Sciences, Dow University of Health Sciences, Karachi 74200, Pakistan; qazirehman@hotmail.com; 6 Department of Oral Pathology, Bahria University Dental College, Karachi 74400, Pakistan; afsheenmaqsood@gmail.com; 7 Department of Community Dentistry, Faculty of Dentistry, Taif University, P.O. Box 11099, Taif 21944, Saudi Arabia; sakeena@tudent.edu.sa; 8 Prosthodontics Unit, School of Dental Sciences, Health Campus, Universiti Sains Malaysia, Kubang Kerian 16150, Kelantan, Malaysia; 9 Department of Prosthodontics, Altamash Institute of Dental Medicine, Karachi 75500, Pakistan; 10 Department of Polymer & Petrochemical Engineering, NED University of Engineering & Technology, Karachi 75270, Pakistan; drkausarali@hotmail.com; 11 Department of Conservative Dental Sciences, College of Dentistry, Prince Sattam Bin Abdulaziz University, P.O. Box 173, Al-Kharj 11942, Saudi Arabia; ma.mustafa@psau.edu.sa; 12 Periodontics, Department of Preventive Dentistry, College of Dentistry, Jouf University, Sakaka 72345, Saudi Arabia; 13 Oral Medicine & Radiology, Department of Oral & Maxillofacial Surgery & Diagnostic Sciences, College of Dentistry, Jouf University, Sakaka 72345, Saudi Arabia; dr.anil.kumar@jodent.org (A.K.N.); drkcs.omr@gmail.com (K.C.S.)

**Keywords:** restorative dentistry, microleakage, class II open sandwich technique, resin-based restorative materials

## Abstract

A vital feature of conservative dentistry is the adhesion of the restorative material to the tooth structure for restoration of the tooth substance lost due to dental decay, trauma, or dental imperfections. In a perfect world, a restorative material should generate a lasting adhesion by bonding the restoration with tooth tissues. The ingress of micro-organisms, oral fluids, molecules, and ions through microscopic spaces due to faulty adhesion between restoration and tooth structure is known as microleakage. This study is focuses on the evaluation of adhesive failures between the restorative materials. In the past, studies have focused more on the bonding potential of a restorative material with the tooth surface. Therefore, there is need to carry out a study that compares the microleakage between resin-based restorative materials in a sandwich manner with and without the intermediate bonding layer after immersion in 2% methylene blue dye at different time intervals. The restorative materials used were composite Ceram X Mono plus (DENTSPLY) and Z350 (3M ESPE), Vitremer resin modified glass ionomer cement (RMGIC) (3M ESPE), smart dentine replacement SDR (3M ESPE), Bond NT (DENTSPLY), and Universal Bond (3M ESPE). A light emitting diode (LED) was used to cure the specimens. Artificial saliva was used as a storage medium for the specimens. Thermocycling of specimens was carried out at 500 cycles/30 s and 1000 cycles/30 s. The world health organization (WHO) grading tool for microleakage was used to analyze fluid ingress in the specimens through disclosing by 2% methylene blue dye. The statistical analysis was carried out with one-way analysis of variance (ANOVA) and Tukey post hoc test, keeping the level of significance at *p* ≤ 0.05. In Grade 0 = 85 samples, Grade 1 = 10 samples, Grade 2 = 7 samples, Grade 3 = 16 samples, and in Grade 4 = 2 samples were identified. This study describes that no microleakage was observed in SDR and resin composite groups as compared to Vitremer and resin composite groups.

## 1. Introduction

Since the invention of dental composites in 1960, they have been modified in the accomplishment of appearance and durability [1]. An important function of a rtorative material is to adhere to the dentine when the enamel is lost due to trauma, caries, or dental treatment [2,3]. The ingress of micro-organisms, oral fluids, molecules, and ions through microscopic cracks due to faulty adhesion between restoration and tooth structure is known as microleakage [4,5]. This can cause increased sensitivity, recurrent caries, pulpitis, and tooth staining [6]. Microleakage in restorative materials could be a consequence of polymerization shrinkage, thermal contraction, water absorption, and mechanical stresses [7].

The methods to reduce microleakage involve various restorative methods (direct or indirect) and curing methods such as the incremental technique [8]. The resin-based restoration shows polymerization shrinkage [9]. Amongst resin restorations, the flowable composite produces decreased polymerization shrinkage, due to lower filler content [10]. Regardless of all scientific advancements, polymerization shrinkage remains the major weakness of the composite resins. The contraction produced by it disrupts the bond with cavity walls and this is a major reason for filling dislodgement, specifically in proximal Class II deep cavities [11,12,13]. One of the techniques suggested for overcoming the class II secondary caries is a “Sandwich Technique” of composite resins and glass ionomer material [14].

McLean and Wilson proposed the concept of open and closed sandwich techniques for class II cavities to overcome the problem of polymerization shrinkage [15]. In this procedure, dentine is replaced by GIC and composite resins replace the enamel part of the tooth. The GIC cement remained uncovered for the release of fluoride at the cervical part of the cavity to reduce the occurrence of dental caries. The purpose of using GIC as a base was also to reduce the amount of resin material. The properties of both restorative materials are combined in a way to decrease caries incidence, enhance chemical adhesion to the tooth structure, release fluoride, longevity, and aesthetics [16,17,18,19]. Other than the conventional GIC, the resin-modified glass ionomer cement (RMGIC), and smart dentine replacement (SDR) can also be used as a base material in the sandwich technique. The RMGIC has a dual ability to bond chemically with the tooth, and also micro mechanically linked to bulk filling composite restorative materials [20]. Moreover, SDR has an increased depth of cure of up to 4 mm thickness. Additionally, it possesses less shrinkage stresses, and increased flowability [21]. This study focuses on analysis of bonding between restorative materials used in an open sandwich technique of class II model cavities.

The current study will provide insight to observe the problem of microleakage precisely. In the past, microleakage and total bonding was evaluated between the material and the tooth structure, but not between the two different materials as in case of sandwich technique. The addition of this study in scientific literature will aid a deep insight on the reasons resin-based restorations fail. The null hypothesis of the study states that “there is no microleakage between the two resin-based restorative materials in a sandwich manner, with and without the intermediate bonding layer after immersion in 2% methylene blue dye at different time intervals”.

The purpose of this study was to compare the microleakage between resin-based restorative materials in a sandwich manner with and without the intermediate bonding layer after immersion in 2% methylene blue dye at different time intervals.

## 2. Materials and Methods

### 2.1. Study Design, Setting, and Duration

This was an in-vitro, experimental study. The samples were made at Dow University of Health Sciences, OJHA campus, Karachi, Pakistan. Thermocycling (PX2, Massachusetts, USA) testing was carried out at OJHA campus. The testing under stereo microscope (Motic DMW 143, PAL System, Hong Kong, China) was done in the Metallurgy department at Nadirshaw Edulji Dinshaw (NED) University of Engineering & Technology, Karachi, Pakistan. The study was completed in one year after the approval of project.

### 2.2. Sampling Technique and Sample Size Estimation

The purposive sampling technique was adopted in this study according to the predefined sample selection criteria. The sample size was estimated following the study of Lawrence et al. [22]. The Open-epi software was used for this purpose. Considering the 95% confidence interval with a 5% margin of error, the power of the test was 80%. The total sample size estimated was 120 specimens, and each group consisted of 10 specimens.

### 2.3. Materials and Sample Selection Criteria

The following materials were included in the study (Table 1):

1. Composite Z350 (3M ESPE, Minnesota United States).
2. Resin Modified Glass Ionomer (3M ESPE Minnesota United States).
3. Ceram X Mono plus (DENTSPLY, York, Pennsylvania, United States).
4. Smart Dentine Replacement, SDR (DENTSPLY, York, Pennsylvania, United States).
5. Bond NT (DENTSPLY, York, Pennsylvania, United States).
6. Universal Bond (3M ESPE, Minnesota United States).

The curing time recommended by the manufacturer was followed for the polymerization of samples in the sandwich manner. Only the LED cured specimens were included in the study. The samples which were broken or contained cracks and air bubbles were not included. Additionally, the incompletely cured samples were also not included in the experiments.

### 2.4. Specification and Fabrication of Samples

**Figure 1:**
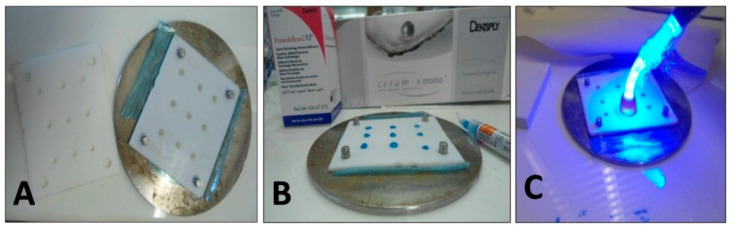
(**A**–**C**): Pictorial description of specimen’s fabrication. (**A**) Mold filled with base material in first sheet. (**B**) Phosphoric acid gel applied on base material. (**C**) Composite filled in second sheet of Teflon sheet, added on top of the first sheet and material cured with LED light.

**Figure 2:**
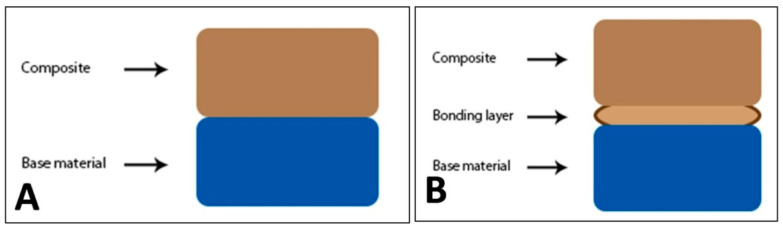
(**A**,**B**): Schematic presentation of the materials interposition. (**A**) Cylindrical specimens in two parts, lower part represents base material and upper part represents the bulk restorative composite (Group A). (**B**) Cylindrical sample in two parts, lower part represents base material and upper part represents the bulk restorative composite, with a bonding adhesive layer in between (Group B).

A specially fabricated mold of polytetrafluoroethylene (Teflon) was used for the preparation of samples. Samples were cylindrical (4 mm diameter and 4 mm height) and followed ISO 4049 for dental composites [22]. Each cylindrical sample consisted of two materials; each material was 2 mm thick as per ISO 10650 [23] as shown in Figure 1 and Figure 2. The mold was designed for mimicking class II restoration; it consists of a metallic base and two Teflon sheets containing nine holes of 4-mm diameter, whereas each sheet was 2 mm in width. The lining material (Vitremer or SDR) was mixed with plastic instrument and packed in one Teflon sheet, cured with LED light for 40 s. Second Teflon sheet was placed on top of the first sheet. The mold was designed in a way that the nine holes of both Teflon sheets overlay each other. Then, the composite material was filled with or without bond application, in the second Teflon sheet and cured for 40 s with LED unit. Group-A had a base material Vitremer or SDR. After curing it was etched for 20 s with 37% phosphoric acid gel, washed for 10 s, and lastly dried for 5 s. After placement of second Teflon sheet, the holes were filled with bulk filling material (Z350 or Ceram X). On the contrary, the Group-B followed the same method with additional application of adhesive material (Vitrebond plus or Single Bond Universal) between base materials and the bulk materials. The samples were then removed from the mold.

The specimens were then stored in an incubator at 26 °C/48 h for completion of the polymerization process. The specimens were placed in dark brown bottles, labeled for each group containing artificial saliva (pH = 6.7). After incubating, the specimens were thermocycle in small tubes filled with artificial saliva to prevent drying during the thermocycling process. It is a method of simulating the temperature variation of the oral cavity at 5–55 °C as per ISO standard 11405 [24].

After taking out small tubes from thermocycler, specimens were removed, dried (with tissue paper), and sealed (two-thirds portion) using two coats of nail varnish. The remaining third portion left uncoated was the dye penetration side (DPS).

Before sectioning, the specimens were dipped in 2% methylene blue dye solution for 24 h (buffered at pH = 7), then washed properly in running tap water. Ultra-thin (22.2 mm × 0.3 mm, ceramic disc) in a micro-motor was used to section the specimens longitudinally in between the DPS, into two halves. The specimens were observed under the Stereomicroscope between magnifications of 10–40× for microleakage observations.

### 2.5. Grouping of the Specimens and Thermocycling Time

**Figure 3:**
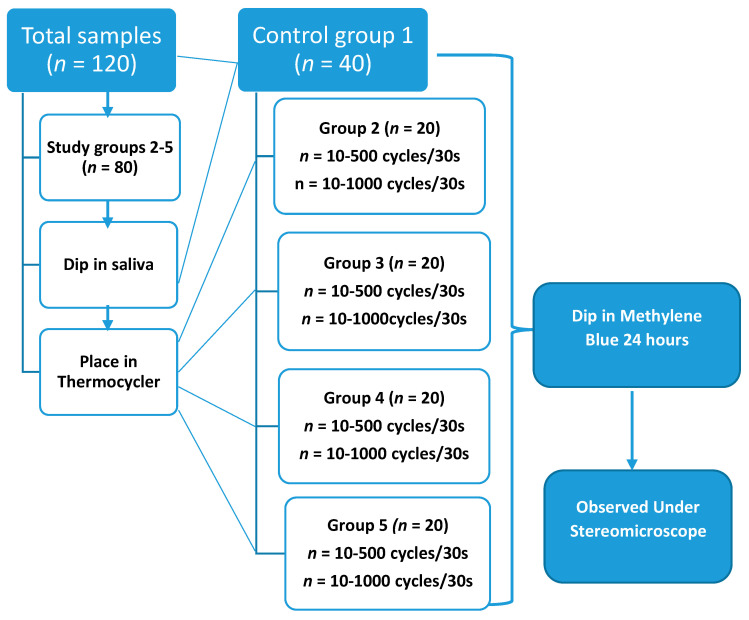
Flow diagram of experimental study.

The following were the three groups with sub-groups included in study (Figure 3):

Group 1: The Control groups which was not subjected to thermocycling.

Group 2:

A = RMGIC (VITREMER) + Z350 composite.
B = RMGIC (VITREMER) + BOND + Z350 composite

Group 3:

A = SDR+ CERAM X composite.
B = SDR+ BOND + CERAM X composite

Group 4:

A = VITREMER + CERAM X composite.
B = VITREMER + BOND + CERAM X composite

Group 5:

5A = SDR+ Z350 composite.
5B = SDR +BOND +Z350 composite

All Group A: specimens were thermocycle at 500 cycles/30 s, whereas the Group B; specimens were thermocycle at 1000 cycles/30 s.

### 2.6. Microleakage Identification Tool

The WHO Microleakage Index tool was used in this study, which can be identified by standard number (ISO/TS 11405:2003) [25,26].

### 2.7. Statistical Analysis

The data were analyzed by Kruskal-Wallis and ANOVA test. The post-hoc analysis (Tukey Test) was carried out to compare microleakage between the groups at three different levels: control, 500 cycles per 30 s, and 1000 cycles per 30 s.

## 3. Results

### 3.1. Pair-Wise Analysis of Microleakage Grading Index by WHO

The distribution of WHO microleakage grading index adopted in this study is described in Table 2. The groupwise analysis of the grading index is shown in Table 3. Each of the five study groups were divided into group A and group B.

Out of the total 34 specimens from the control were classified as grade zero (0.0 mm leakage), there were 15 specimens of group 1A and 19 specimens of group 1B. Furthermore, three specimens of the second group had grade zero together with one specimen of group 2A and two from group 2B. Likewise, all 20 specimens were categorized as grade zero in the third group, including 10 of group 3A and 10 from group 3B. Nine samples of 4B group were grade zero. Nevertheless, 10 specimens of group 5A and nine specimens of group 5B from the fifth group were grade zero.

In addition, two specimens of group 1A from the control group were included as grade one (up to 0.5 mm leakage). Moreover, one specimens of group 2A and five specimens from group 2B were classified as grade one. In the 4B and 5B groups, only one specimen from each group was found to be in grade one.

Grade two (up to 1 mm leakage) was associated with one specimen from each group 1A and 1B from control respectively. One specimen from the 2A group and two specimens from the 2B group, were grade two, while two specimens from 4A group showed grade two microleakage.

Likewise, grade three (up to 2 mm leakage) categories consisted of two specimens from group 1A and the control group. Five specimens of group 2A and one sample of group 2B from the second group, and lastly eight specimens from the 4A group were associated with grade 3. The grade four microleakage (≥2 mm) was found in only two specimens from the 2A group.

### 3.2. Distribution of Microleakage Values in Control and Experimental Groups after Thermocycling

**Figure 4:**
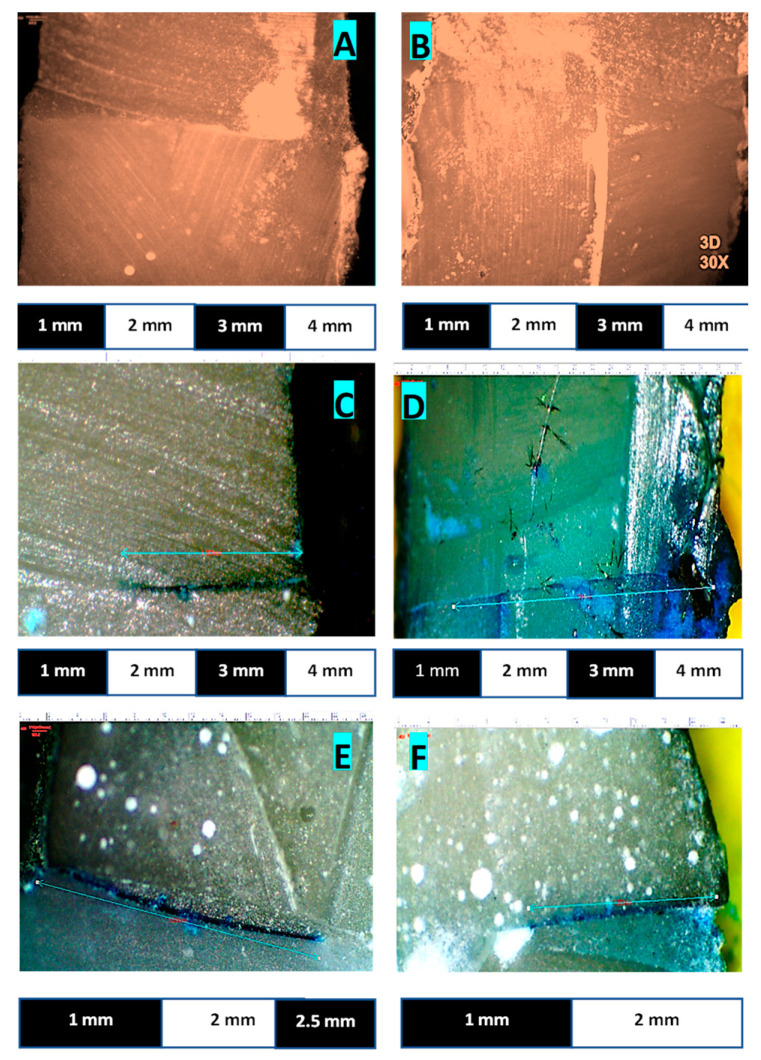
(**A**–**F**): (**A**) Stereomicroscope image taken at 25× stereomicroscope from Group 3A Control level; (**B**) Stereo image taken at 30× stereomicroscope from Group 3B Control level; (**C**) Stereo Image taken at 45× stereomicroscope from Group 2A 500 cycles/30 s; (**D**) Stereo image taken at 45× stereomicroscope from Group 2A 1000 cycles/30 s; (**E**) Stereo image taken at 45× stereomicroscope from Group 4A 500 cycles/30 s; (**F**) Stereo image taken at 45× stereomicroscope from Group 4A 1000 cycles/30 s.

The mean values of a control group without thermocycling and study groups with the thermocycling procedure are shown in Table 4 and the pictorial description is given in Figure 4. The highest mean values in the control group were observed in 2A, 0.82 ± 0.86; whereas similar microleakage values were found in 2A, 3A, and B, as well as in groups 4 and 5. The mean difference values of specimens were found to be zero in third and fifth groups with an insignificant *p*-value > 0.99.

In the second and fourth groups, the mean difference was 0.82 and 0.18 respectively. There was a significant difference (*p* = 0.05) in the values of group 2. However, no significant difference (*p* = 0.14) was noted in group 4 microleakage values. 

The mean difference of second group at 500 cycles per 30 s was 0.75 and fourth group were 1.16, with a significant *p*-value of 0.016 and 0.01 respectively. Furthermore, the second, fourth and fifth group at 1000 cycles per 30 s has shown a mean difference of 0.95, 1.15, and 0.06 with a non-significant *p*-values of 0.17, 0.17, and 0.32 respectively. 

### 3.3. Proportionality between A Groups

Table 5 is describing the proportionality of A subgroups in control, and after 500 cycles per 30 s, and 100 cycles per 30 s thermocycling procedures. No difference was found in microleakage among materials in the control group. Although at 500 cycles per 30 s, a difference was noted when the second and fourth groups were compared with third and fifth groups. A difference in values was also noted when the second group was compared with third and fourth at 1000 cycles per 30 s. The mean values of second and fourth control group were 0.82 ± 0.86 and 0.18 ± 0.25 respectively. Considering, 500 cycles per 30 s, the results indicated that in second group the mean value was 1.25 ± 0.13 and in the fourth group it was 1.22 ± 0.40. Furthermore, at 1000 cycles per 30 s, the second group showed a mean value of 1.26 ± 1.23 and group four had a mean value of 1.15 ± 0.30. Additionally, the third and fifth groups had a zero value in the control, and after thermocycling.

### 3.4. Proportionality among B Groups

Table 6 is showing the proportionality of B subgroups in control, 500 cycles per 30 s and 1000 cycles per 30 s. After thermocycling at 500 cycles per 30 s, a remarkable difference was found when second group was compared with all other groups. The second group revealed a highest mean value of 0.50 ± 0.36, whereas at 1000 cycles per 30 s, the mean value was 0.31 ± 0.32. Additionally, in the fourth group the mean value was the least 0.07 ± 0.15 at 500 cycles per 30 s, whereas, in the fifth group the mean value was 0.11 ± 0.06 at 500 cycles and 0.24 ± 0.14 at 1000 cycles per 30 s.

## 4. Discussion

The current study evaluated the success and failure of Class II open sandwiched technique restoration, by investigating the microleakage at the resin–resin interface. The stereomicroscope was used to observe eight variable combinations of base and bulk restorative materials. No significant difference was found in the microleakage values of the third and fifth groups, both groups showed minimal or zero leakage scores. The SDR material was used as base material in both the groups where no microleakage was observed, indicating a better bonding ability of SDR with resin composite material. Whereas a significant microleakage scores was found in group two and four in this study. The Vitremer and composite bond was found weaker. Thus, the null hypothesis that “There is no microleakage between the two resin-based restorative materials in a sandwich manner, with and without the intermediate bonding layer after immersion in 2% methylene blue dye at different time intervals” was rejected.

Nicola and Scotti, reported a similar outcome with SDR in their study, the only difference was in the methodology, they used extracted molar specimens, filled with restorative materials [27]. Furthermore, the presence of urethane di-methacrylate and di-methacrylate resin could be the reason for no leakage in SDR, both resins enhance chemical adhesion with tooth structure. Sadeghi et al. used thermocycle samples at 1500 cycles, fuchsine dye was applied to identify the microleakage. He applied flowable composite and SDR in the specimens. An adequate adhesion was found primarily due to the use of flowable resins which has reduced modulus of elasticity that decrease stresses within the restoration. A better physical characteristic, easy handling, less polymerization shrinkage, and improved curing depth of SDR could also be the reason for the favorable outcome [28].

El-Safty et al. [29] and Leprince J.G. et al. [30] found the same results with flowable composites. They further suggested that ‘swelling’ behavior of particular bulk fill composites could also be one of the reasons for reducing the microleakage. The authors checked the properties of the composite by Raman spectroscopy and Vickers hardness testing [30]. In the current study, absolutely no leakage specifies improved bonding ability of the Ceram X and SDR in groups 1, 3, and 5 which was evaluated after being thermocycled at 500 cycles per 30 s and 1000 cycles per 30 s at room temperature. Schirrmeister et al. found similar results in an in-vivo study using Ryge’s criteria. It was proposed that Ceram-X had an improved clinically proven marginal seal [31]. Similarly, Ahmadi et al. [32], used class V cavities in intact molar to analyze microleakage through fuchsin dye with a similar methodology and they found no microleakage in nano-ceramic composite at the cervical margins. The absence of microleakage with Ceram X was also found by Eden et al. [33] with similar methods. In a clinical study conducted by Schmidt et al. [34], utilizing scanning electron microscopy showed a similar finding with Ceram X composites. Thus, it is proven by the literature that a superior sealing ability of Ceram X composites is one of the reasons for reduced microleakage over time. On the contrary, Sing et al. [35] found that Ceram-X composite was associated with microleakage. This finding is comparable with our study in terms of methodology, the difference could be due to the variation in the base materials [35]. Likewise, Bogra et al. [36] used extracted teeth with thermocycling at 2–5 °C for 1500 cycles and observed leakage at cemento-enamel junction and occlusal margins while using Ceram X nano-composite in class 2 cavities. Kermanshah et al. [37] encountered microleakage at the gingival margin with Ceram X nano-ceramic composite comparatively higher than Filtek silorane composite. They used composite materials in class I and II cavities on extracted teeth to measure leakage by the same regime as adopted in the present study. Agrawal et al. [38] also described a considerably low microleakage scores in nano-ceramic and silorane composite materials with a ribboned fiber base material. In another study by Owen B et al. [39], an increased microleakage was noted in nano-composite resins in contrast to micro-hybrid composites while used in class 5 cavities, when sectioned and immersed in 1% methylene blue dye. Because of the contradictory results related to Ceram X, this study focused on a combination of materials in a sandwich manner. Nonetheless, in this study, microleakage was found in Ceram X and Vitremer combination groups 2 and 4.

The etch and rinse protocol is the gold standard in dental adhesive systems to date, this study followed the same protocol, for an adequate micro-mechanical retention of composite resin and base materials [40]. The Ceram X and Z350 have proven adhesive efficacy in past studies of class 2 cavities [41]. The application of SDR as a base material in third and fifth groups showed no significant difference in this study.

The proportionality of second and fourth groups (Vitremer and Z350; Vitremer, 3M, and Ceram X) in this study revealed microleakage. It was observed in all subgroups (with and without bond, under 500 and 1000 cycles), but the extent of leakage was different among the groups studied. The control groups 2A and 4A also showed microleakage. This could be due to the internal gaps in the restoration as described Owen B et al. [39] and Spencer et al. [42].

In this study, the bond between the materials was checked after thermocycling to reflect the aging effect of the restoration. Gerdolle et al. [43] claimed in a study that bonds between resin-based materials can be enhanced due to thermal expansion and contraction processes within matured restorations.

In this study, the SDR and Vitremer comparison as a base material revealed unsatisfactory sealing with Vitremer, even with an inherent property of chemical adhesion to enamel and dentin. The brittle nature of RMGIC (Vitremer) could be the cause of this distress by the formation of cracks with thermal changes [44].

Feilzeret et al. [45] described that the microleakage may result in composite restoration by water sorption specifically in the unfilled resin type. They observed minimum leakage in nano-RMGI and nano-composite while used in a sandwich manner. El-Ashiry et al. [46] used the same material in class V cavities of primary molars with 0.5% fuchsin dye to identify leakage under stereomicroscope while, AB Malik et al. [47] used in class II cavities. Both authors found decreased leakage at the occlusal margins compared to the gingival margin in nano-RMGI, compared to conventional RMGIC. Beznos et al. [48] proposed that RMGIC decreases volumetric shrinkage of resin-based restorations by 41%. This finding did not match with the results of the present study. In this study, greater microleakage was observed in groups having RMGIC—i.e., the second and fourth groups. Polymerization shrinkage, missing elastic deformation, increased viscosity and porosities could be the cause of increase microleakage in RMGIC [49]. The other reason of microleakage could be dual setting characteristics of RMGIC compared to SDR which is light cure [50].

This study adopted the dye penetration method for finding microleakage. Despite visible flaws in dye penetration, no evidence of errors is reported yet about this method [51,52,53]. The procedure is validated and easy to perform even in a simple laboratory setup [54]. The 2% methylene blue dye is simple to utilize, it has increased water solubility and quick diffusion in cracks or imperfections in a substance [55]. The methylene blue is considered to have a better dye penetration than butyric acid [56,57]. Other methods such as dye and radioisotope penetration are precise and produce 82% accuracy in microleakage detection [58,59]. Due to these properties reported in the literature, the methylene blue dye method was selected to be used in this study [59].

The ingress of fluid or microleakage in restoration is an indicator of bond failure between them that can occur due to various reasons such as polymerization shrinkage, materials incompatibility, and thermal changes in a particular material with time [57]. Numerous methods such as matched adhesive use, incremental technique application, and control of C-factor by balancing the number of bonded and unbonded tooth surfaces are recommended in the literature to overcome microleakage under restorations [60]. This study proposes the application of suitable base material (SDR) prior to the filling of bulk filled resin composite material to achieve adequate bond between materials as well as with the tooth structure. However, further investigation in the form of clinical trials is recommended to compare the efficacy of resin-based material combinations in long-term use, and to better understand the outcome of restorative materials available.

## 5. Conclusions

This study was carried out to analyze the microleakage between different combinations of resin-based materials. Within the limitations of the study, following conclusion can be drawn:

1. SDR with Ceram X and with Z350 exhibited no leakage, indicating that SDR would have better bonding affinity with resin composites at different time intervals.
2. The bonding of Vitremer with the resin composite was weak.

## Figures and Tables

**Table 1:** Material used in this study.

Materials	Type	Composition	Manufacturer
Vitremer	Resin-based material (RMGIC)	Tri-cure glass ionomer powder-fluoroaluminosilicate glass. Micro-encapsulated potassium per sulfate and ascorbic acid as catalyst system. Glass ionomer liquid-polycarboxylic acid, HEMA, and photo initiators. Primer and finishing gloss.	3M ESPE, Minnesota United States
Z350	Polymethylmethacrylate based resin composite	Bis-GMA, UDMA, TEGDMA, Bis-EMA resins, PEGDMA, and zirconia fillers.	3M ESPE, Minnesota United States
SDR	Resin-based base material	SDR™ patented urethane di-methacrylate resin Di-methacrylate resin Di-functional diluents Barium and Strontium Aluminofluorosilicate glasses Photo initiating system colorants	DENTSPLY, York, Pennsylvania, United States,
Ceram X Mono+	Polymethylmethacrylate based resin composite	Organically modified ceramic and nanoparticles fillers, as used in Prime & Bond NT combines with conventional glass fillers of 1 µm	DENTSPLY York, Pennsylvania, United States
Prime & Bond^®^ NT™	Nano-technology dental adhesive	Di- and trimethacrylate resins Penta (dipentaerythritol Penta-acrylate monophosphate) Nano fillers-amorphous silicon dioxide Photoinitiators Stabilizers Cetylamine hydro fluoride Acetone Caulk 34% tooth conditioner gel Phosphoric acid Highly dispensed silicon dioxide Colorant Water	DENTSPLY, York, Pennsylvania, United States
Single Bond Universal	Adper™ Scotch bond™ multi-purpose adhesive	MDP phosphate monomer Dimethacrylate resins HEMA Vitrebond™ copolymer Filler Ethanol Water Initiators Silane	3M ESPE, Minnesota United States

**Table 2:** Distribution of microleakage analysis grades adopted in this study.

Grade	Description
Grade 0	0.0 mm
Grade 1	up to 0.5 mm micro leakage
Grade 2	up to 1 mm micro leakage
Grade 3	up to 2 mm micro leakage
Grade 4	≥2 mm

**Table 3:** Detailed pair-wise analysis of microleakage grading index by WHO.

Row Labels	* Group 1		Group 2		Group 3		Group 4		Group 5		Grand Total
	A	B	A	B	A	B	A	B	A	B	
Grade 0	15	19	1	2	10	10	0	9	10	9	85
Grade 1	2	0	1	5	0	0	0	1	0	1	10
Grade 2	1	1	1	2	0	0	2	0	0	0	7
Grade 3	2	0	5	1	0	0	8	0	0	0	16
Grade 4	0	0	2	0	0	0	0	0	0	0	2
Grand Total	20	20	10	10	10	10	10	10	10	10	120

A = Without bond; B = With bond; WHO = World Health Organization; * Group 1 was control.

**Table 4:** Comparison of microleakage values in study groups 2, 3, 4, and 5 after thermocycling.

Material		Control Group	500 Cycles/30 s	1000 Cycles/30 s
Group 2	(A)	0.82 ± 0.86	1.25 ± 0.13	1.26 ± 1.22
	(B)	0.00	0.50 ± 0.36	0.30 ± 0.31
	Mean difference (*p*-value)	0.82 (0.05 *)	0.75 (0.016 *)	0.95 (0.17)
Group 3	(A)	0.00	0.00	0.00
	(B)	0.00	0.00	0.00
	Mean difference (*p*-value)	0 (>0.99)	0 (>0.99)	0 (>0.99)
Group 4	(A)	0.18 ± 0.24	1.22 ± 0.39	1.14 ± 0.30
	(B)	0.00	0.06 ± 0.14	0.00
	Mean difference (*p*-value)	0.18 (0.14)	1.16 (0.01 *)	1.15 (0.17)
Group 5	(A)	0.00	0.00	0.00
	(B)	0.00	0.00	0.62 ± 0.14
	Mean difference (*p*-value)	0 (>0.99)	0 (>0.99)	0.06 (0.32)

* Significant at *p* ≤ 0.05, A = Without bond, B = With bond.

**Table 5:** Proportionality of groups A.

Material	Control	500 Cycles/30 s	1000 Cycles/30 s
Group 2	0.82 ± 0.86	1.25 ± 0.13	1.26 ± 1.23
Group 3	0	0	0
Group 4	0.18 ± 0.25	1.22 ± 0.40	1.15 ± 0.30
Group 5	0	0	0

Mean and Standard deviation of groups 2, 3, 4, and 5A.

**Table 6:** Proportionality of groups B.

Material	Control	500 Cycles/30 s	1000 Cycles/30 s
Group 2	0	0.50 ± 0.36	0.31 ± 0.32
Group 3	0	0	0
Group 4	0	0.07 ± 0.15	0
Group 5	0.11 ± 0.24	0	0.06 ± 0.14

Mean and Standard deviation of groups 2, 3, 4, and 5B.

## Data Availability

The data presented in this study are available on request from the corresponding author.

## Abbreviations

**S. No**	**Abbreviation**	**Full Name/Description**
1	ADA	American Dental Association
2	ANOVA	One-way analysis of variance
3	Bis-GMA	Bisphenol A-glycidyl methacrylate
4	CDC	Centre for Disease Control
5	CTE	Co-efficient of thermal expansion
6	DPS	Dye penetration site
7	HEMA	Hydroxyethyl methacrylate
8	ISO	International Standard Organization
9	LED	Light emitting diode
10	META	Methacryloxyethyl trimellmellitate anhydride
11	RBC	Resin-based composites
12	RMGIC	Resin modified glass ionomer cement
13	SDR	Smart dentine replacement
14	TEGDMA	Triethylene glycol dimethacrylate
15	Teflon	Polytetrafluoroethylene
16	UDMA	Urethane dimethacrylate
